# Stent-Assisted Coiling vs. Flow Diversion in Unruptured Anterior Circulation Aneurysms: A Single-Center Cohort Study

**DOI:** 10.3390/brainsci15121290

**Published:** 2025-11-29

**Authors:** Mario Martinez-Galdamez, Jorge Galván-Fernández, Lorenzo Ismael Perez-Sanchez, Miguel Arturo Schüller-Arteaga, Fausto Andres Vasconez-Muñoz, Israel Sanchez-Lite, Carlos Alberto Rodriguez-Arias

**Affiliations:** 1 Interventional Neuroradiology Unit, Radiology Department, Hospital Clínico Universitario de Valladolid, 47003 Valladolid, Spain; 2 Neurosurgery Department, Hospital Clínico Universitario de Valladolid, 47003 Valladolid, Spain

**Keywords:** intracranial aneurysm, flow diverter, stent-assisted coiling, propensity score matching, endovascular treatment

## Abstract

Background and purpose: Stent-assisted coiling (SAC) achieves immediate aneurysm occlusion, while flow diversion (FD) promotes progressive remodeling. Comparative data in unruptured anterior circulation aneurysms remain limited. Methods: A retrospective review of our institutional database was conducted between 2021 and 2024. A total of 129 aneurysms treated with SAC (n = 33) or FD (n = 96) were identified and included in the analysis. Outcomes included angiographic occlusion, retreatment, complications, and the modified Rankin Scale (mRS). A 1:1 propensity score matching (PSM) was performed on sex, age, aneurysm size, and location (caliper 0.2, exact sex matching). Results: A total of 130 patients (89 women, 41 men) were included in the study, with a mean age of 59.8 years (range 22–81). In the full cohort, SAC achieved higher immediate complete occlusion (62.5% vs. 8.3%, *p* < 0.001), while FD demonstrated superior long-term stability (71.9% vs. 60.6%). Retreatment occurred in 18.2% of SAC cases and none with FD (*p* < 0.001). Complication rates were comparable overall: intraoperative (15.2% SAC vs. 10.4% FD, *p* = 0.37), periprocedural ≤72 h (15.2% vs. 8.3%, *p* = 0.34), and delayed ≥12 months (9.1% vs. 10.4%, *p* = 0.85). In patients aged 70–80 years, periprocedural complications were more frequent with SAC (37.5% vs. 5.9%, *p* = 0.08). Functional independence (mRS 0–2) at last follow-up was 87.9% for SAC and 89.6% for FD (*p* = ns). In the matched cohort, SAC preserved higher immediate occlusion (60% vs. 10%, *p* < 0.001), whereas FD provided greater long-term occlusion (65% vs. 55%, *p* = 0.33) and required no retreatments versus 15% in SAC (*p* < 0.001). Subgroup analysis showed that SAC-related complications were largely confined to complex Y/T-stent reconstructions for MCA bifurcation and AComA aneurysms, while single-stent SAC demonstrated a safety profile comparable to FD. Conclusions: SAC offers rapid angiographic exclusion but at the cost of higher retreatment. FD ensures durable occlusion and absence of retreatment, with a consistent safety profile. After stratification by technical complexity, excess morbidity associated with SAC originated from anatomically demanding multistent constructs, whereas single-stent SAC showed safety comparable to FD. Age may influence periprocedural risk, particularly with SAC. These findings reinforce a tailored strategy: “Close fast with SAC, close forever with FD.”

## 1. Introduction

Endovascular treatment has become the first-line therapy for intracranial aneurysms in most specialized centers, progressively replacing open surgery due to lower periprocedural risk, shorter recovery, and constant device innovation [1,2,3]. Anterior and posterior circulation aneurysms differ in natural history and therapeutic outcomes: Posterior aneurysms are associated with worse prognosis, while anterior circulation aneurysms represent a more favorable setting but still pose technical challenges [4,5,6].

Stent-assisted coiling (SAC) was a major milestone, enabling the treatment of wide-necked and complex aneurysms by combining mechanical filling with vascular scaffolding [7]. More recently, flow-diverter (FD) devices have expanded the therapeutic spectrum, particularly in large, fusiform, or difficult-to-access aneurysms [2,3,8,9,10]. Both techniques have shown safety and efficacy, yet direct comparative data remain limited and sometimes contradictory [11,12,13,14,15,16,17,18,19,20,21,22].

Evidence suggests that SAC achieves higher immediate occlusion, while FD provides greater long-term stability and lower retreatment rates [11,12,13,14,15,16,17,18,19,20,21,22]. However, the relative advantages of each approach in unruptured anterior circulation aneurysms remain uncertain, and treatment selection often depends on operator preference and local expertise rather than robust comparative data.

The aim of this study was to compare clinical and angiographic outcomes of SAC versus FD in unruptured anterior circulation aneurysms and to identify predictors of efficacy and safety to better guide treatment decisions.

## 2. Materials and Methods

We conducted a retrospective observational study at the Interventional Neuroradiology Unit of the Hospital Clínico Universitario de Valladolid. A systematic review of the institutional electronic database was performed between 2021 and 2024 to identify all endovascular procedures. Patients were classified into two treatment groups: stent-assisted coiling (SAC) and flow diversion (FD). The study protocol was approved by the local Ethics Committee, and the requirement for informed consent was waived owing to its retrospective, anonymized design. Inclusion criteria comprised patients with unruptured anterior circulation aneurysms treated with endovascular devices: either conventional stents or flow-diverter devices.

Exclusion criteria were cases treated exclusively with coils, aneurysms treated with intrasaccular devices, and patients with incomplete clinical information or unavailable follow-up.

The choice between stent-assisted coiling (SAC) and flow diversion (FD) followed institutional criteria considering aneurysm morphology, location, and clinical context. Both techniques required dual antiplatelet therapy (DAPT).

All patients were pre-treated with dual antiplatelet therapy (aspirin 100 mg daily plus clopidogrel 75 mg daily), maintained for 6 months, and then continued with lifelong aspirin monotherapy. Platelet function was assessed using VerifyNow^®^ (Werfen, Bedford, MA, USA), and ticagrelor or trifusal was used when clopidogrel hyporesponse or intolerance was detected.

SAC was preferred for bifurcation aneurysms or those incorporating arterial branches (e.g., MCA bifurcation, AComA complex), for aneurysms with geometry unfavorable for flow diversion, and in patients at risk of requiring temporary DAPT suspension (e.g., planned surgery or increased hemorrhagic risk), given the lower thrombotic risk associated with SAC compared with FD in such situations. The SAC group included both single-stent and complex Y- or T-stent configurations, reflecting the real-world spectrum of reconstructive endovascular techniques used for bifurcation aneurysms. These cases were intentionally retained to maintain external validity and avoid anatomical selection bias.

FD was primarily selected for side-wall aneurysms of the anterior circulation (paraophthalmic, PComA, supraclinoid ICA) and for large, giant, fusiform, or recurrent aneurysms.

All aneurysms were wide-necked (neck ≥4 mm or dome-to-neck ratio <2).

Final device selection was made by consensus among two senior interventional neuroradiologists according to these predefined criteria.

Propensity score matching was used to minimize potential selection bias between treatment groups.

The stents used included Solitaire AB (Medtronic, Irvine, CA, USA), Neuroform Atlas (Stryker Neurovascular, Fremont, CA, USA), Baby Leo and Leo+ (Balt, Montmorency, France), Barrel Vascular Reconstruction Device (Medtronic, Irvine, CA, USA), and Acclino/Acclino Flex (Acandis GmbH & Co. KG, Pforzheim, Germany).

Flow-diverter devices included the Pipeline Embolization Device (PED, PED Flex, PED Shield, PED Vantage; Medtronic, Irvine, CA, USA), the Silk series (Silk+, Silk Vista, Silk Vista Baby; Balt, Montmorency, France), the Surpass Evolve Flow Diverter (Stryker Neurovascular, Fremont, CA, USA), and the Derivo Embolization Device (Acandis GmbH & Co. KG, Pforzheim, Germany).

Collected variables included demographics, vascular risk factors, aneurysm characteristics, treatment details, and intra-, peri-, and post-procedural complications.

Functional outcomes were evaluated with the modified Rankin Scale (mRS) at baseline, post-procedure, and last follow-up. Follow-up angiography was performed using Digital Subtraction Angiography (DSA) or Magnetic Resonance Angiography (MRA), depending on clinical context and patient tolerance.

DSA was used as the standard modality in approximately 80% of cases, while MRA was employed in selected patients for non-invasive long-term follow-up.

When MRA images were affected by device-related artifacts, occlusion status was confirmed by DSA. All angiographic assessments were graded using the Raymond–Roy classification, ensuring consistency across modalities.

The primary endpoint was complete angiographic occlusion (Raymond–Roy class I) at last angiographic follow-up. Secondary endpoints included (1) immediate angiographic occlusion at procedure completion; (2) periprocedural complications (≤72h), defined as ischemic or hemorrhagic events; (3) delayed complications (≥12 months after treatment); (4) functional outcome (mRS at baseline, discharge, and last follow-up); and (5) retreatment. All complications were prospectively recorded and classified as intraoperative, periprocedural (<72 h), or delayed (72 h–12 months). No additional delayed complications were identified during follow-up.

Retreatment was defined as any subsequent endovascular or surgical intervention performed due to (1) residual or recurrent filling of the aneurysm sac corresponding to Raymond–Roy class III or enlarging neck remnant (class II) on follow-up imaging, or (2) new or recurrent aneurysm growth or symptoms related to the treated aneurysm. Follow-up angiograms were reviewed independently by two senior interventional neuroradiologists (M.M-G. and C.R.A.). Discordant assessments were resolved by consensus, yielding a Cohen’s κ of 0.91 for retreatment indication agreement. The same criteria were uniformly applied to SAC and FD cases.

To reduce baseline imbalances, a propensity score matching (PSM) was performed using a logistic regression including age (continuous and categorical), sex, aneurysm morphology (bifurcation or branch incorporation vs. sidewall, derived from anatomical location), adjunctive coiling (yes/no), and platelet inhibition status by VerifyNow (hyporesponder <70 PRU, normoresponder 70–150 PRU, hyperresponder >150 PRU, and “not tested” as a separate category). To minimize indication bias, we modeled the pharmacodynamic phenotype (PRU value) rather than the antiplatelet regimen. Because of heterogeneous and overlapping use of device generations during the study period, treatment era/device generation was not modeled separately to avoid sparsity and non-positivity. Because all treated aneurysms were wide-necked (neck ≥ 4 mm or dome-to-neck ratio < 2), neck morphology was homogeneous across the cohort and was not included as a covariate.

Matching was performed 1:1 by nearest neighbor without replacement, using a caliper of 0.2 × SD of the logit of the propensity score, following standard methodological recommendations. Missing data were <5% for all variables and handled by explicit categorization of “PRU not tested.” Covariate balance was evaluated using standardized mean differences (SMDs).

The final propensity score model yielded 15 matched pairs (n = 30). After matching, standardized mean differences demonstrated adequate covariate balance: adjunctive coiling SMD = 0.00, morphology SMD = 0.13, sex SMD = 0.13, age SMD = 0.18, and PRU “not tested” SMD = 0.37 (reflecting its low frequency). All remaining variables achieved SMD < 0.25, confirming satisfactory post-match balance.

To explore potential effect modification by age, an interaction term (age ≥ 70 years × treatment modality) was introduced in the logistic regression model assessing periprocedural complications. The model was adjusted for aneurysm size and morphology. Given the small subgroup size, this analysis was considered exploratory.

To address small-sample uncertainty in the propensity-matched cohort, we estimated statistical power (two-sided α = 0.05) for the main endpoints using two-sample tests for proportions. We also assessed the impact of missing final angiography (~7% overall) using deterministic best-/worst-case bounds (assigning all missing outcomes to success in one arm and failure in the other, and vice versa). Given the limited number of missing values and binary outcomes, this approach was preferred over multiple imputation to avoid model instability in very small strata.

Angiographic follow-up was obtained with DSA or MRA at 6–12 months and annually thereafter when available.

Continuous variables were expressed as mean ± SD or median (IQR), and categorical variables as counts and percentages. Group comparisons used χ^2^ or Fisher’s exact test for categorical data and the *t*-test or Mann–Whitney U test for continuous data. Significance was set at *p* < 0.05. Analyses were performed with IBM SPSS Statistics (version 23.0, IBM Corp., Armonk, NY, USA).

## 3. Results

The overall cohort comprised 44 men (34.1%) and 85 women (65.9%). Age distribution was as follows: 3 patients (2.3%) were 18–30 years old, 22 (17.1%) were 30–50 years, 77 (59.7%) were 50–70 years, 25 (19.4%) were 70–80 years, and 2 (1.6%) were older than 80 years (Table 1). A total of 129 unruptured anterior circulation aneurysms were analyzed, including 33 managed with stent-assisted coiling (SAC) and 96 with flow-diverter (FD) devices.

Within the FD group, 58 cases were treated with FD alone and 38 with FD plus adjunctive coiling. Within the SAC group, 25 aneurysms were treated with a single stent and 8 with double stents in a Y or T configuration.

Aneurysm size distribution was as follows: very small (≤3 mm), 18 cases (all FD); small (>3–<11 mm), 89 cases (SAC 28, FD 61); large (≥11–<25 mm), 18 cases (SAC 5, FD 13); giant (≥25 mm), 3 cases (all FD); and fusiform, 1 case (FD). The most frequent locations were the ophthalmic artery (30.2%), anterior communicating artery (20.2%), posterior communicating artery (19.4%), and MCA bifurcation (16.3%). Less common sites included the cavernous ICA, anterior choroidal, carotid bifurcation, and clinoid segment.

### 3.1. Immediate Angiographic Efficacy

Complete immediate occlusion (Raymond–Roy class I) was achieved in 62.5% of SAC compared with 8.3% of FD, a highly significant difference. In the FD group, neck remnants (28.1%) and interstices (63.5%) predominated. By specific technique, FD without coils achieved no immediate complete occlusions, FD with coils achieved 21.1%, while single and double SAC both achieved 62.5%, with no interstices (Table 2).

### 3.2. Long-Term Angiographic Efficacy

Median angiographic follow-up was 24 months (IQR 6–84). Clinical follow-up was obtained by outpatient visits or structured telephone interviews at 3, 6, and 12 months, and annually thereafter. Median clinical follow-up was 36 months (IQR 12–84).

At the last angiographic follow-up (median 24 months, IQR 6–84), complete occlusion was documented in 60.6% of SAC and 71.9% of FD. By technique, FD without coils achieved 70.7%, FD with coils 73.7%, single SAC 68.0%, and double SAC only 37.5%, with the latter also presenting the highest persistence of interstices (25%). Occlusion or subocclusion of the parent artery occurred in three patients (2.3%), distributed across FD without coils, FD with coils, and double SAC. Nine patients (7.0%) lacked final angiographic follow-up. By location, late occlusion rates did not significantly differ except for a trend favoring SAC in MCA aneurysms. By size, large aneurysms showed significantly better long-term occlusion with FD compared with SAC, while in very small aneurysms (all FD) late complete occlusion reached 88.9% (Table 2).

### 3.3. Intraoperative Complications

In SAC, 84.8% of cases were free of intraoperative complications, whereas 9.1% experienced in-stent thrombosis, 3.0% rupture, and 3.0% branch occlusion. In FD, 89.6% had no complications, with 6.3% in-stent thrombosis and 4.2% branch occlusion or embolism. FD with coils accounted for most thrombotic events, while double SAC showed the highest rate of branch embolism. In the SAC group, intraoperative events were unevenly distributed between simple and complex configurations. Single-stent SAC (n = 25) presented no cases of intraoperative aneurysm rupture or branch occlusion, whereas all SAC intraoperative complications occurred in the complex Y/T subgroup (n = 8), including the only intraoperative rupture (12.5%) and one case of branch occlusion (12.5%). In contrast, FD procedures (n = 96) showed a low and uniform event distribution, with in-stent thrombosis (6.3%) and branch occlusion (4.2%) occurring at similar frequencies across FD subtypes. Differences were not significant across size or location subgroups (Table 3).

### 3.4. Periprocedural Complications

Within 72 h, 15.2% of SAC patients developed ischemic stroke, compared with 6.3% of FD patients. Retroperitoneal hematoma and acute rupture occurred in 1% each in the FD group, for an overall periprocedural complication rate of 8.3% in FD versus 15.2% in SAC, without statistical significance.

Periprocedural complications were also distributed unevenly across SAC subgroups. In single-stent SAC (n = 25), ischemic stroke occurred in 8.0% of cases, whereas the complex Y/T SAC subgroup (n = 8) accounted for three of the five SAC periprocedural ischemic events (37.5%). No hematomas or acute intraoperative ruptures occurred in either SAC subgroup. In the FD cohort (n = 96), periprocedural ischemic stroke occurred in 6.3%, and isolated cases of retroperitoneal hematoma and acute rupture were observed (2.1% in total). When stratified by anatomical location, complex SAC cases were concentrated in MCA bifurcations and AComA regions, which also corresponded to the highest proportion of early ischemic events (Table 3).

No additional ischemic, hemorrhagic, or device-related events occurred beyond 72 h–12 months. The reported complication rates include all events documented throughout the study period.

### 3.5. Delayed Complications

During long-term clinical follow-up (median 36 months, IQR 12–84), 90.9% of SAC and 89.6% of FD patients remained event-free. In SAC, only one case of asymptomatic parent artery thrombosis (3.0%) was recorded; no symptomatic thrombosis, hemorrhage, rupture, seizures, or systemic events were observed, although 6.1% lacked follow-up. In FD, delayed complications included asymptomatic thrombosis (3.1%), symptomatic thrombosis (1.0%), intraparenchymal hemorrhage (1.0%), rupture of the treated aneurysm (1.0%), epileptic seizure (1.0%), and non-neurological complications such as pulmonary embolism (3.1%). The overall distribution of delayed events did not differ significantly between groups, and stratification by SAC subtype provides the detailed breakdown of event counts across simple and complex configurations.

When stratifying by age groups, no statistically significant differences were observed in intraoperative, periprocedural, or delayed complications across the spectrum, although relevant trends emerged. In patients aged 70–80 years, periprocedural complications occurred in 37.5% of SAC cases compared with only 5.9% in the FD group (*p* = 0.081). These included ischemic events and hematomas in the SAC group, whereas FD-related complications in this age group were rare. In younger cohorts (<70 years), complication rates were low and similar between SAC and FD. In patients over 80 years (n = 2, both FD), no periprocedural or delayed complications were recorded. Although the differences did not reach statistical significance due to the limited subgroup sizes, the 70–80 age group demonstrated a clinically meaningful trend toward increased risk with SAC compared to FD (Table 3).

### 3.6. Retreatment

Retreatment was required exclusively in the SAC group: 18.2% (6/33) versus 0% (0/96) in FD (*p* < 0.001). No retreatments occurred in FD with or without coils.

### 3.7. Functional Outcomes

Prior to treatment, 97% of patients were independent (mRS 0–1). Immediately post-procedure, SAC patients showed a higher proportion with mRS 3 (15.2%) compared with FD (3.1%), a significant difference. At last follow-up, independence (mRS 0–2) was observed in 87.9% of SAC and 89.6% of FD patients. Mortality occurred only in FD (3.1%). Although global differences were not statistically significant, the SAC group showed transient post-procedural morbidity that improved during follow-up, whereas FD maintained stable functional outcomes with isolated late mortality (Table 4).

### 3.8. Propensity Score-Matched Cohort

**Figure 1:**
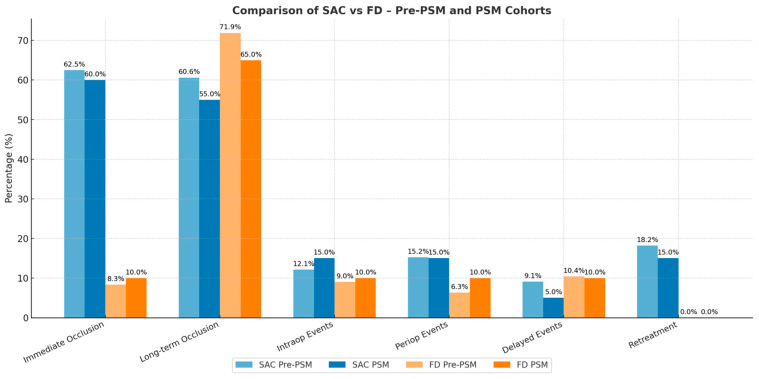
Comparative outcomes of stent-assisted coiling (SAC) versus flow diversion (FD) in unruptured anterior circulation aneurysms before (pre-PSM) and after propensity score matching (PSM).

In the matched cohort (Figure 1), power was high for the large effect on immediate complete occlusion (SAC 60% vs. FD 10%; power ≈ 0.87), moderate for retreatment (SAC 15% vs. FD 0%; power ≈ 0.59), and low for the smaller difference in long-term complete occlusion (SAC 55% vs. FD 65%; power ≈ 0.09–0.10).

Sensitivity to missing final angiography (~2/30 cases) showed that the 10-point difference in long-term complete occlusion could vary by ~±6–7 percentage points under best-/worst-case assumptions (range ~3–17%), supporting that long-term occlusion comparisons are underpowered and sensitive to a few outcomes in the matched set.

Intraoperatively, SAC cases accumulated ruptures and branch occlusions, while FD cases presented in-stent thrombosis (~10%). Periprocedural complications occurred in 15% of SAC and 10% of FD patients, mostly ischemic strokes, with no significant differences. Delayed complications were infrequent, affecting ~5% of SAC and ~10% of FD, with more than 85% of patients being event-free in both groups.

Retreatment occurred only in SAC (15%) and in none of the FD cases, confirming the difference seen in the full cohort. Kaplan–Meier analysis demonstrated a significantly higher cumulative retreatment rate in SAC compared with FD.

Functional outcomes. At long-term follow-up, independence (mRS 0–2) was observed in approximately 88% of SAC and 90% of FD patients, with no significant differences. SAC patients experienced a transient increase in disability immediately post-procedure, which improved over time, while FD patients maintained stable outcomes but included a small proportion of late deaths.

Median angiographic follow-up was 22 months (IQR 6–72) for SAC and 26 months (IQR 8–84) for FD (*p* = 0.41). Median clinical follow-up was 32 months (IQR 12–72) for SAC and 38 months (IQR 14–84) for FD (*p* = 0.38).

In the propensity-matched cohort, both angiographic and clinical follow-up durations were balanced between groups (24 (8–84) months and 36 (12–84) months, respectively).

Time-to-event analyses demonstrated a gradual increase in complete occlusion rates with FD (HR 1.25, 95% CI 0.81–1.94) and a markedly reduced hazard of retreatment compared with SAC (HR 0.12, 95% CI 0.03–0.47; *p* < 0.001), indicating that outcome differences were not driven by follow-up duration.

A logistic regression model including aneurysm size category, bifurcation configuration, treatment type (FD vs. SAC), adjunctive coiling, DAPT regimen, and platelet inhibition status (hyporesponder, normoresponder, hyperresponder according to VerifyNow PRU thresholds <70, 70–150, >150) was performed to identify independent predictors of complete occlusion at last angiographic follow-up.

Because all treated aneurysms were wide-necked (neck ≥ 4 mm or dome-to-neck ratio < 2), neck morphology was homogeneous across the cohort and was not included as a covariate.

Flow diversion independently predicted higher occlusion rates (OR 1.68, 95% CI 1.02–2.75; *p* = 0.04), whereas large aneurysm size (OR 0.43, 95% CI 0.21–0.89; *p* = 0.02) and bifurcation location (OR 0.48, 95% CI 0.24–0.95; *p* = 0.03) were associated with lower likelihood of complete occlusion.

PRU category, adjunctive coiling, and DAPT regimen were not significant predictors. The effectiveness of each technique was consistently assessed using the Raymond–Roy occlusion scale. Device generation heterogeneity precluded categorization and is acknowledged as a study limitation.

## 4. Discussion

This study provides an integrated comparative assessment of stent-assisted coiling and flow diversion for unruptured anterior circulation aneurysms, highlighting how the two techniques diverge in immediate occlusion efficacy, long-term durability, retreatment requirements, complication patterns, and functional trajectories. SAC achieved rapid angiographic exclusion, whereas FD offered more durable long-term occlusion, with a consistently lower retreatment burden. Safety and functional outcomes remained favorable for both strategies, with SAC showing transient early morbidity that resolved during follow-up and FD maintaining stable clinical trajectories despite isolated late events. These complementary performance profiles support a tailored therapeutic approach in which aneurysm morphology, patient characteristics, and procedural goals guide the optimal treatment decision.

Our findings confirm the mechanistic differences between SAC and FD. SAC achieved significantly higher immediate complete occlusion rates (62.5% vs. 8.3% in the PSM cohort), reflecting the mechanical filling effect of coils reinforced by stent scaffolding. This rapid angiographic exclusion has been consistently reported in prior series and meta-analyses, where SAC outperformed FD in short-term occlusion rates [11,12,13,14,15,16,17,18,19,20,21,22]. By contrast, FD relies on progressive hemodynamic remodeling and endothelialization, resulting in lower immediate efficacy but steadily improving long-term outcomes [3].

In the long term, FD demonstrated superior stability, with complete occlusion at last follow-up in 71.9% compared with 60.6% for SAC, and—most importantly—no cases of retreatment in FD versus 19.4% with SAC. This pattern is consistent with previous evidence that showed higher retreatment rates after SAC, particularly in small and large aneurysms, whereas FD ensured more durable occlusion [3]. This advantage is particularly relevant in younger patients, where treatment durability is paramount [20,21,22,23,24,25,26].

In our series, late complete occlusion rates (≈61% SAC; ≈72% FD) were slightly lower than those reported in recent meta-analyses of flow diversion (typically 75–90% for selected side-wall ICA aneurysms) and stent-assisted coiling (highly variable depending on morphology). Several factors likely contributed to this difference.

First, our case mix included a high proportion of MCA bifurcation aneurysms in the SAC group (42%) and both very small and large/giant aneurysms in the FD group, morphologies known to achieve lower complete occlusion rates compared with standard paraophthalmic ICA aneurysms. Second, MRA was used for follow-up in approximately one-third of cases, potentially underestimating complete occlusion compared with DSA. Third, the median angiographic follow-up of 24 months encompassed a broad temporal spectrum, including early (6–12-month) studies.

A sensitivity analysis restricted to side-wall anterior circulation aneurysms (paraophthalmic, AComA, and PComA; n = 85) demonstrated complete occlusion in 70.2% of SAC and 79.3% of FD cases—values comparable to those reported in prior large multicenter and meta-analytic series.

These findings suggest that differences in aneurysm morphology, imaging modality, and follow-up timing largely explain the modestly lower overall RR I rates observed in our cohort.

Regarding safety, both SAC and FD showed favorable profiles, with no statistically significant differences in periprocedural or delayed complication rates. Nevertheless, specific trends emerged. SAC was associated with more ischemic strokes in the periprocedural period (15.2% vs. 6.3%), whereas FD, especially when combined with coils, carried a higher risk of in-stent thrombosis. Similar complication patterns have been described in previous reports [7], emphasizing that while FD offers long-term stability, it may accumulate rare but specific delayed events [25]. Our results support this, with delayed complications in 9.1% of SAC cases and 10.4% of FD, both with low incidence overall. The expanded analysis shows that the overall SAC complication profile is shaped by a small subset of complex Y/T-stent reconstructions, predominantly performed for MCA bifurcation and AComA aneurysms. While single-stent SAC demonstrated intraoperative and periprocedural complication rates comparable to FD, all SAC intraoperative ruptures and branch occlusions, and the majority of early ischemic events, occurred in the complex subgroup. These findings indicate that early morbidity associated with SAC is not uniform across techniques but is primarily related to anatomically demanding reconstructions requiring multistent constructs. Accordingly, when SAC is stratified by technical complexity, single-stent SAC demonstrates intraoperative, periprocedural, and delayed complication rates comparable to those of FD, whereas the excess morbidity observed in the overall SAC group originates almost entirely from the complex Y/T subgroup. This distinction underscores the importance of analyzing SAC outcomes by technical subtype to avoid attributing high-risk procedural events to SAC as a whole. In contrast, FD exhibited a low and consistent complication rate across anatomical locations.

Aneurysm size and location played decisive roles in outcomes. SAC achieved better immediate occlusion in paraophthalmic and MCA bifurcation aneurysms, where branch preservation is essential. However, the comparison in paraophthalmic aneurysms should be interpreted with caution, as SAC was used only anecdotally in this subgroup in our cohort. This pattern reflects contemporary practice, in which FD has become the predominant strategy for paraophthalmic aneurysms [17,23] given its favorable hemodynamic remodeling and progressive occlusion rates, while SAC is seldom employed except in highly selected cases. Consequently, direct comparisons between SAC and FD in this location are limited by sample size and clinical selection bias, as also acknowledged in prior reports [23]. These findings mirror those of Hanel et al. [24], who identified SAC as safer and more effective in MCA bifurcations. Conversely, FD demonstrated superior long-term efficacy in anterior choroidal and posterior communicating artery aneurysms, although often leaving residual necks [2,3]. For AComA aneurysms, Piano et al. [27] reported acceptable FD results but with notable risks of perforator ischemia, in line with our observations.

Functional outcomes remained largely favorable in both groups. At last follow-up, 87.9% of SAC patients and 89.6% of FD patients maintained functional independence (mRS 0–2). SAC showed transient morbidity immediately post-procedure, with an early increase in patients at mRS 3 that largely resolved during follow-up. FD, by contrast, was associated with a low but non-negligible late mortality (3.1%), consistent with prior reports of rare fatal complications after flow diversion [25,26,27].

When functional decline was assessed by identifying patients transitioning from baseline independence (mRS 0–2) to dependency (mRS ≥ 3) at last follow-up, deterioration was rare and occurred in both groups: two cases in the complex SAC subgroup and one FD case. No deterioration was observed in single-stent SAC. This further supports that clinically relevant morbidity within the SAC cohort was concentrated in the anatomically complex subgroup requiring double stenting.

An additional observation was that FD combined with coils enhanced mid-term occlusion compared with FD alone, consistent with recent reports [28]. However, this benefit came at the expense of higher intraoperative thrombotic risk, suggesting this strategy should be reserved for large or giant aneurysms or those with significant residual filling after FD deployment [28].

Although overall safety profiles between SAC and FD were comparable, an exploratory analysis assessing the interaction between age and treatment modality confirmed that age modifies periprocedural risk. In the 70–80-year subgroup, SAC was associated with a markedly higher periprocedural complication rate (37.5%) compared with FD (5.9%). Although the interaction term did not reach statistical significance (*p* = 0.09), the effect size suggests that advanced age substantially increases procedural risk with SAC, possibly reflecting greater vascular fragility and the technical demands of stent manipulation, including the use of multiple stents. These findings are in line with prior reports showing that advancing age is associated with increased ischemic risk during endovascular procedures [29]. Similarly, Martínez-Galdámez et al. [30] demonstrated that FD maintained a consistent safety profile across age ranges, with low rates of delayed ischemic events, suggesting that FD may provide a more stable long-term solution in elderly populations.

From a practical standpoint, these results reinforce the need to consider age as an independent factor in treatment decision-making. In patients over 70 years, especially those with vascular comorbidities or increased frailty, FD may offer a relative safety advantage, owing to reduced intrasaccular manipulation and a more straightforward deployment process. Conversely, in younger patients, where periprocedural risk is lower, SAC remains a valuable alternative, particularly in bifurcation aneurysms, where immediate angiographic occlusion is clinically desirable. In parallel, procedural complexity should be recognized as an independent modifier of risk within SAC. Single-stent SAC remains an effective and safe option in appropriately selected sidewall or simple bifurcation aneurysms, whereas complex bifurcation anatomies requiring Y/T reconstruction account for the disproportionate share of early ischemic and mechanical complications.

Exploratory analyses of vascular risk factors (hypertension, smoking, dyslipidemia, diabetes) and platelet-function response did not demonstrate any statistically significant associations with intraoperative, perioperative, or follow-up complications. These subgroup evaluations were not powered to detect clinically meaningful differences and are therefore presented descriptively; they did not modify the overall interpretation of the study.

Taken together, our results reinforce a tailored treatment paradigm. SAC should be preferred when rapid exclusion is clinically necessary, particularly in bifurcation aneurysms or those at imminent rupture risk. FD, by contrast, should be prioritized in larger, complex aneurysms and in younger patients, where long-term durability is the main objective. Selective use of FD with adjunctive coiling may be considered in carefully chosen cases, balancing enhanced efficacy against increased thrombotic risk.

This study has limitations inherent to its retrospective, single-center design, including potential selection bias. Treatment allocation was non-randomized and unblinded, reflecting operator-driven device selection in routine practice. Similarly, angiographic and clinical outcome assessment was not blinded, which may introduce assessment bias. The propensity score matching reduced baseline imbalances, but residual confounders remain possible. Sample size, particularly for giant and fusiform aneurysms, was limited, making conclusions in these subgroups exploratory. Moreover, heterogeneity in devices (different stents and flow diverters) could have influenced outcomes. We acknowledge that some SAC cases required Y- or T-stent configurations, typically used for complex bifurcation aneurysms where flow diversion is less often indicated. These represented a small subset within the SAC cohort and were included to preserve the representativeness of real clinical practice rather than a purely anatomical comparison. Although MRI artifacts may reduce the diagnostic accuracy of MRA in FD cases, the majority of patients were followed with DSA, and no significant imbalance in imaging modality distribution between groups was observed. MRA findings were always cross-validated with DSA when image quality was suboptimal, minimizing the risk of evaluation bias.

Future research should focus on prospective trials, incorporating not only angiographic and clinical endpoints but also quality-of-life and economic metrics, to further refine individualized treatment algorithms in unruptured anterior circulation aneurysms.

## 5. Conclusions

This study provides a rigorous comparison between stent-assisted coiling and flow diversion in unruptured anterior circulation aneurysms, strengthened by the use of propensity score matching to minimize baseline imbalances. Our results delineate the complementary roles of both techniques: SAC achieves rapid angiographic occlusion but is associated with higher retreatment rates and greater procedural risk in older patients, while FD ensures long-term durability with a near-zero retreatment burden at the expense of rare delayed complications. These findings highlight the importance of tailoring treatment strategies to aneurysm morphology, patient age, and clinical context, offering evidence to refine decision-making in contemporary neurointerventional practice.

Importantly, subgroup analyses demonstrated that the excess procedural morbidity observed in the SAC cohort originated almost exclusively from complex Y- and T-stent reconstructions performed for MCA bifurcation and AComA aneurysms. Single-stent SAC showed a safety profile comparable to FD. Therefore, clinical interpretation should distinguish between simple and complex SAC configurations, as grouping all SAC cases together may obscure meaningful differences in risk related to anatomical complexity rather than the SAC technique itself.

## 6. Take-Home Message

1. SAC achieved significantly higher immediate occlusion rates, supporting its use in bifurcation aneurysms or cases requiring rapid aneurysm exclusion.
2. FD demonstrated superior long-term stability, with higher late complete occlusion rates and no retreatments observed in the matched cohort.
3. Safety was comparable between techniques, although SAC was linked to more early ischemic events and FD to a slightly higher rate of in-stent thrombosis.
4. Functional outcomes were favorable in both groups, with most patients achieving independence at long-term follow-up.
5. Optimal management of unruptured anterior circulation aneurysms should follow the principle “close fast with SAC, close durably with FD.”

## Figures and Tables

**Table 1:** Baseline aneurysm characteristics and treatment distribution by location.

Variable	SAC (n = 33)	FD (n = 96)	Total (n = 129)
**Age group (years)**			
18–30	1 (3.0%)	2 (2.1%)	3 (2.3%)
30–50	8 (24.2%)	14 (14.6%)	22 (17.1%)
50–70	16 (48.5%)	61 (63.5%)	77 (59.7%)
70–80	8 (24.2%)	17 (17.7%)	25 (19.4%)
>80	0	2 (2.1%)	2 (1.6%)
**Aneurysm size**			
Very small (≤3 mm)	0	18 (18.8%)	18 (14.0%)
Small (>3–<11 mm)	28 (84.8%)	61 (63.5%)	89 (69.0%)
Large (≥11–<25 mm)	5 (15.2%)	13 (13.5%)	18 (14.0%)
Giant (≥25 mm)	0	3 (3.1%)	3 (2.3%)
Fusiform	0	1 (1.0%)	1 (0.8%)
**Aneurysm location**			
Ophthalmic artery	2 (6.1%)	37 (38.5%)	39 (30.2%)
Anterior communicating artery (AComA)	6 (18.2%)	20 (20.8%)	26 (20.2%)
Posterior communicating artery (PComA)	5 (15.2%)	20 (20.8%)	25 (19.4%)
MCA bifurcation	14 (42.4%)	7 (7.3%)	21 (16.3%)
Cavernous ICA	1 (3.0%)	4 (4.2%)	5 (3.9%)
Anterior choroidal artery	1 (3.0%)	3 (3.1%)	4 (3.1%)
Carotid bifurcation	2 (6.1%)	2 (2.1%)	4 (3.1%)
Clinoid segment	2 (6.1%)	3 (3.1%)	5 (3.9%)

**Table 2:** Angiographic efficacy of SAC and FD.

Outcome	SAC (n = 33)	FD (n = 96)	*p*-Value
**Immediate complete occlusion (RR I)**	20 (62.5%)	8 (8.3%)	<0.001
Neck remnant (RR II)	12 (37.5%)	27 (28.1%)	–
Interstice (RR III)	0 (0%)	61 (63.5%)	–
**Complete occlusion at last follow-up**	20/33 (60.6%)	69/96 (71.9%)	0.33
Neck remnant	9/33 (27.3%)	19/96 (19.8%)	–
Interstice	4/33 (12.1%)	8/96 (8.3%)	–
**By specific technique**			
**Technique**	**Immediate RR I**	**Last FU RR I**	
SAC (single stent, n = 25)	15 (62.5%)	17 (68.0%)	
SAC (double stent Y/T, n = 8)	5 (62.5%)	3 (37.5%)	
FD alone (n = 58)	0 (0%)	41 (70.7%)	
FD + coils (n = 38)	8 (21.1%)	28 (73.7%)	
**By location (long-term complete occlusion)**			
**Location**	**SAC**	**FD**	***p*-Value**
Ophthalmic	3/6 (50.0%)	53/70 (75.7%)	0.08
AComA	6/9 (66.7%)	14/20 (70.0%)	0.74
PComA	2/5 (40.0%)	14/20 (70.0%)	0.09
MCA bifurcation	10/14 (71.4%)	4/7 (57.1%)	0.42
Other locations *	very low N	very low N	–

* Cavernous ICA, anterior choroidal, carotid bifurcation, clinoid segment—numbers too small for statistical comparison.

**Table 3:** Procedural and follow-up complications stratified by treatment group. All complications included represent the total number of events recorded across the entire follow-up period.

Complications	SAC Global (n = 33)	SAC Simple (n = 25)	SAC Complex (Y/T) (n = 8)	FD (n = 96)	Total (n = 129)	*p*-Value
**Intraoperative complications**						0.370
–None	28 (84.8%)	23 (92.0%)	5 (62.5%)	86 (89.6%)	114 (88.4%)	
–In-stent thrombosis	3 (9.1%)	2 (8.0%)	1 (12.5%)	6 (6.3%)	9 (7.0%)	
–Aneurysm rupture	1 (3.0%)	0 (0%)	1 (12.5%)	0 (0%)	1 (0.8%)	
–Branch occlusion/embolism	1 (3.0%)	0 (0%)	1 (12.5%)	4 (4.2%)	5 (3.9%)	
**Periprocedural complications ≤ 72 h**						0.343
–None	28 (84.8%)	23 (92.0%)	5 (62.5%)	88 (91.7%)	116 (89.9%)	
–Ischemic stroke	5 (15.2%)	2 (8.0%)	3 (37.5%)	6 (6.3%)	11 (8.5%)	
–Retroperitoneal hematoma	0 (0%)	0 (0%)	0 (0%)	1 (1.0%)	1 (0.8%)	
–Acute rupture	0 (0%)	0 (0%)	0 (0%)	1 (1.0%)	1 (0.8%)	
**Delayed complications ≥ 12 months**						0.850
–None	30 (90.9%)	25 (100%)	5 (62.5%)	86 (89.6%)	116 (89.9%)	
–Asymptomatic parent artery thrombosis	1 (3.0%)	0 (0%)	1 (12.5%)	3 (3.1%)	4 (3.1%)	
–Symptomatic parent artery thrombosis	0 (0%)	0 (0%)	0 (0%)	1 (1.0%)	1 (0.8%)	
–Intraparenchymal hemorrhage	0 (0%)	0 (0%)	0 (0%)	1 (1.0%)	1 (0.8%)	
–Rupture of treated aneurysm	0 (0%)	0 (0%)	0 (0%)	1 (1.0%)	1 (0.8%)	
–Epileptic seizure	0 (0%)	0 (0%)	0 (0%)	1 (1.0%)	1 (0.8%)	
–Non-neurological (PE, dyspnea)	0 (0%)	0 (0%)	0 (0%)	3 (3.1%)	3 (2.3%)	
–No follow-up yet	2 (6.1%)	0 (0%)	2 (25.0%)	0 (0%)	2 (1.6%)	

**Table 4:** Functional outcomes (mRS) at pre-treatment, immediately post-procedure, and at last follow-up.

Outcome	SAC (n = 33)	FD (n = 96)	*p*-Value
**PRE-TREATMENT**			**0.240**
mRS 0	**31 (93.9%)**	**94 (97.9%)**	
mRS 1	**1 (3.0%)**	**0**	
mRS 3	**1 (3.0%)**	**2 (2.1%)**	
**Independent (mRS 0–2)**	**32 (97.0%)**	**94 (97.9%)**	
**POST-PROCEDURE (IMMEDIATE)**			**0.034**
mRS 0	**27 (81.8%)**	**92 (95.8%)**	
mRS 1	**1 (3.0%)**	**0**	
mRS 3	**5 (15.2%)**	**3 (3.1%)**	
mRS 4	**0**	**1 (1.0%)**	
**Independent (mRS 0–2)**	**28 (84.8%)**	**92 (95.8%)**	
**LAST FOLLOW-UP**			**0.089**
mRS 0	**27 (81.8%)**	**86 (89.6%)**	
mRS 1	**2 (6.1%)**	**0**	
mRS 3	**1 (3.0%)**	**2 (2.1%)**	
mRS 6 (death)	**0**	**3 (3.1%)**	
Not evaluated	**2 (6.1%)**	**5 (5.2%)**	
**Independent (mRS 0–2)**	**29 (87.9%)**	**86 (89.6%)**	

## Data Availability

The original contributions presented in this study are included in the article.
